# Assessment of *Aspergillus niger* Strain’s Suitability for Arsenate-Contaminated Water Treatment and Adsorbent Recycling via Bioextraction in a Laboratory-Scale Experiment

**DOI:** 10.3390/microorganisms8111668

**Published:** 2020-10-27

**Authors:** Eva Duborská, Kinga Szabó, Marek Bujdoš, Hana Vojtková, Pavol Littera, Edmund Dobročka, Hyunjung Kim, Martin Urík

**Affiliations:** 1 Institute of Laboratory Research on Geomaterials, Faculty of Natural Sciences, Comenius University in Bratislava, Mlynská dolina, Ilkovičova 6, 84215 Bratislava, Slovakia; eva.duborska@uniba.sk (E.D.); kinga.szabo.agnik@gmail.com (K.S.); marek.bujdos@uniba.sk (M.B.); littera@broz.sk (P.L.); 2 Department of Environmental Engineering, Faculty of Mining and Geology (FMG), Technical University of Ostrava, 17. listopadu 2172/15, 708 00 Ostrava-Poruba, Czech Republic; hana.vojtkova@vsb.cz; 3 Institute of Electrical Engineering, Slovak Academy of Sciences, Dúbravská cesta 9, 841 04 Bratislava, Slovakia; edmund.dobrocka@savba.sk; 4 Department of Mineral Resources and Energy Engineering, Jeonbuk National University, 567, Baekje-daero, Deokjin-gu, Jeonju, Jeonbuk 54896, Korea; kshjkim@jbnu.ac.kr

**Keywords:** arsenic, bioaccumulation, bioextraction, ferric oxyhydroxides, filamentous fungi

## Abstract

In this work, the viability of bioaccumulation and bioextraction processes for arsenic removal from contaminated waters, as well as the recycling of arsenate-treated amorphous ferric oxyhydroxide adsorbent (FeOOH) were evaluated using the common soil microscopic filamentous fungus *Aspergillus niger*. After treating the contaminated arsenate solution (100 mg As L^−1^) with FeOOH, the remaining solution was exposed to the growing fungus during a static 19-day cultivation period to further decrease the arsenic concentration. Our data indicated that although the FeOOH adsorbent is suitable for arsenate removal with up to 84% removal efficiency, the fungus was capable of accumulating only up to 13.2% of the remaining arsenic from the culture media. This shows that the fungus *A. niger*, although highly praised for its application in environmental biotechnology research, was insufficient for decreasing the arsenic contamination to an environmentally acceptable level. However, the bioextraction of arsenic from arsenate-treated FeOOH proved relatively effective for reuse of the adsorbent. Due to its production of acidic metabolites, which decreased pH below 2.7, the fungal strain was capable of removing of up to 98.2% of arsenic from the arsenate-treated FeOOH adsorbent.

## 1. Introduction

The release and mobilization of arsenic in complex geological substrates is a result of various chemical and biological processes that take place on the arsenic-bearing minerals’ surfaces [1]. This primarily includes reductive dissolution and desorption which are regulated by geochemical factors and by the presence and activity of microorganisms [2,3].

In addition to autotrophic iron(III) respiration by bacterial strains [4,5], heterotrophic leaching by filamentous fungi has been suggested as an effective tool for bioextraction of metals and metalloids from iron-rich rocks, minerals and industrial waste products [6,7]. However, bioextraction can also be applied on adsorbents when considering their regeneration after usage in heavy metal removal from contaminated waters. This has been demonstrated by Adams et al. [8], who desorbed zinc from zinc-spiked charcoal using chelating extracellular metabolites of the filamentous fungal strains of *Trichoderma harzianum*, *T. reesei* and *Coriolus versicolor*.

Among filamentous fungi, *Aspergillus niger* has a unique position since it has been one of the most referenced fungal species over the last 40 years [9] and frequently applied in biohydrometallurgical research [10]. Extensive mobilization of arsenic via the interaction of fungal *A. niger* strain’s extracellular metabolites with arsenic-bearing mineral’s surfaces has been previously reported by Kolenčík, et al. [11]. Similarly, our previous publication has shown that *A. niger* is capable of desorbing arsenic from ferric hydrated oxyhydroxides [12]. Hence, we believe that the biologically driven extraction can be applied for regeneration of arsenic loaded iron-based adsorbents when their maximum adsorption capacity has been reached.

The expected effect of naturally occurring bioextraction is an increase in the mobility and concentration of arsenic in the groundwater and, thus, it enhances the arsenic exposure to organisms, including humans. Since arsenic is considered the most significant chemical contaminant in drinking water globally and can cause deleterious effects even at low concentrations [13], the guidelines have decreased its maximum allowable level to 10 µg L^−1^ in various countries [14]. Therefore, the arsenic removal from water bodies is a high priority of environmental health and safety management.

Among others, iron-based adsorbents offer promising results for arsenic removal from waters [15]. Still, their adsorption capacity is limited by the contact time in column systems or equilibrium concentration in batch systems. This greatly reduces the adsorption capacity and allows contaminants to pass through without being immobilized. In this case, the recommended acceptable levels of arsenic are not achieved and therefore, some sort of post-adsorption treatment method for contaminated water must be used.

Recently, bioaccumulation and biosorption have been suggested as viable and promising methods in the treatment of arsenic contaminated waters [16]. However, the evaluation of “viability” of biological water-treatment in conjunction with a traditional chemical and physical approach is virtually non-existent, since it is usually reported for biologically driven remediation separately from standard techniques, even at the laboratory-scaled level [17]. Moreover, it appears that the biological approaches are typically expected to perform well and be advantageous in comparison to non-biological processes, while omitting drawbacks or apparent unsatisfactory performance of biological systems in arsenic removal [18,19].

This lack of information inspired us to perform a complex three-step laboratory-scaled experiment that includes adsorption and bioaccumulation of arsenic, as well as the regeneration of adsorbent using fungal metabolites. The *A. niger* strain was used deliberately, since it is ubiquitous, arsenic-tolerant and commonly found in contaminated soils and sediments [20,21]. Furthermore, it produces large amounts of acidic and chelating agents that contribute to the dissolution of solid materials [22], hence, it is a good candidate for both the bioaccumulation and bioextraction of arsenic. Surprisingly, our results indicated limited applicability of the filamentous fungus, *A. niger*, for remediation of arsenate-contaminated water as a post-treatment method to further decrease the arsenic concentration to an acceptable level. However, natural biodeterioration of arsenate-treated ferric oxyhydroxide adsorbent, which resulted in arsenic bioextraction, showed that the fungus is capable of increasing a sorbent’s reusability in contaminated-water treatment techniques.

## 2. Materials and Methods

### 2.1. Adsorbent Synthesis

The X-ray diffraction (XRD) amorphous phase of hydrated ferric oxyhydroxides (FeOOH) used as an adsorbent in this study was prepared by alkaline (40 g NaOH p.a. ≥99%; Centralchem, Bratislava, Slovakia) precipitation of FeCl_3_ (54.06 g FeCl_3_.6H_2_O p.a. ≥ 99%; Centralchem, Bratislava, Slovakia) in 1 L of deionized water under laboratory conditions. After a 12 h period of stirring at 150 rpm (Unimax 2010, Heidolph, Schwabach, Germany), freshly prepared precipitates were filtered, washed with distilled water, and dried at 80 °C overnight. The synthetized ferric precipitate was stored in a sealed container at room temperature before experiments.

### 2.2. Sorption Experiment

The desired amount (1–8 g L^−1^) of FeOOH, which consists of nanosized akaganeite, goethite, and lepidocrocite [23] was added to Erlenmeyer flasks with a 50 mL solution of arsenate with an arsenic concentration of 100 mg L^−1^. The stock solution of arsenate was prepared from Na_2_HAsO_4_.7H_2_O (ACS reagent, ≥98%; Sigma–Aldrich, Taufkirchen, Germany), which was dissolved using redistilled water. A FeOOH-free run was also tested.

The prepared suspensions were placed onto a horizontal shaker (Unimax 2010; Heidolph, Schwabach, Germany), and agitated at 130 rpm for 24 h in the dark at 25 °C. Thereafter, the suspensions were filtered through a 0.45 µm pore-size mixed cellulose esters (MCE) membrane filter. The filtrates were diluted to 100 mL with redistilled water and there the total content of arsenic was determined by flame atomic absorption spectrometry (F-AAS; Perkin Elmer Model 1100 (Waltham, USA), wavelength 193.7 nm, air-acetylene flame, deuterium background correction, LOQ ~ 0.5 mg L^−1^, the standard expanded uncertainty (k = 2) is 6%) or inductively coupled plasma mass spectrometry (ICP-MS; Thermo Scientific (Waltham, USA) iCap Q in KED (kinetic energy discrimination) mode (He), LOQ ~ 0.005 mg L^−1^, the standard expanded uncertainty (k = 2) is 4%).

### 2.3. Bioaccumulation Experiment

The fungal strain of *Aspergillus niger* CBS 140,837 (hereinafter referred to as *A. niger*) was isolated from mercury-contaminated soil in Slovakia [20] and deposited at the Mycological Laboratory collection at the Plant Science and Biodiversity Center of the Slovak Academy of Sciences. Prior to inoculation, *A. niger* strain was maintained on Sabouraud agar slants (4% *w*/*v*; “for microbiology” grade, HiMedia, Mumbai, India) at 4 °C and spore suspension was prepared from 7-day old culture.

In total, 3 g of Sabouraud dextrose broth (“for microbiology” grade, HiMedia, Mumbai, India) was added to 50 mL of filtrate, which was collected after sorption, and sterilized in a drying oven (UN55; Memmert, Schwabach, Germany) for 2 h at 70 °C. Thereafter, each Erlenmeyer flask was inoculated with *A. niger* spores under aseptic conditions in a laminar flow cabinet (BIO100 type A; Alpina, Treviso, Italy). The inoculated samples were cultivated for 19 days in the dark at 25 °C in a laboratory incubator (Pol-eko Aparatura, Wodzisław Śląski, Poland). After 19 days, the fungal aerial mycelium (only partially submerged into a culture medium) was separated from the surface of the growth solution, rinsed with redistilled water, and air-dried to constant weight. The dry biomass weight was measured. The pH of the spent medium was determined using electrode HI 1230B (Hanna Instruments, Italy). The total arsenic content in the spent culture medium and biomass was determined using F-AAS or ICP–MS [24] after digestion in a microwave system Multiwave 3000 (Anton Paar, Graz, Austria) with a mixture of concentrated acids (HF + HNO_3_ + HClO_4_ + H_2_O_2_; all reagents were of analytical reagent grade or higher quality suitable for trace metal analysis, and were obtained from Centralchem, Bratislava, Slovakia).

### 2.4. Bioextraction (Bioleaching) Experiment

To determine bioextraction efficiency of arsenic from the FeOOH phase collected after the sorption experiment, a one-step extraction using *A. niger* strain was applied. The recovered FeOOH on the membrane filter was placed in a 100 mL Erlenmeyer flask, and sterilized in the laboratory oven at 70 °C for 10 h. The day prior to inoculation with *A. niger* spore suspension, each sample had been sterilized for another 40 min at 95 °C, and 50 mL of sterilized Sabouraud dextrose broth medium was added. After inoculation, the fungus was cultivated for 19 days in the dark at 25 °C in the laboratory incubator. After a 19-day cultivation period, the fungal biomass was collected, rinsed with redistilled water, and air-dried to constant weight. The spent growth medium was filtered through a 0.45 µm pore-size MCE membrane filter to separate the remaining FeOOH phase from the growth medium. The collected FeOOH phases were digested in a microwave system with a mixture of concentrated acids (HF + HNO_3_ + HClO_4_ + H_2_O_2_). This was followed by determination of total arsenic content using ICP–MS or F–AAS [25]. Iron was determined by flame atomic absorption spectrometry (F–AAS, Perkin Elmer Model 1100 (Waltham, USA), wavelength 248.3 nm, air-acetylene flame, deuterium background correction, LOQ ~ 0.02 mg L^−1^, the standard expanded uncertainty (k = 2) is 4%).

## 3. Results

### 3.1. ArsenateAadsorption onto FeOOH

**Figure 1:**
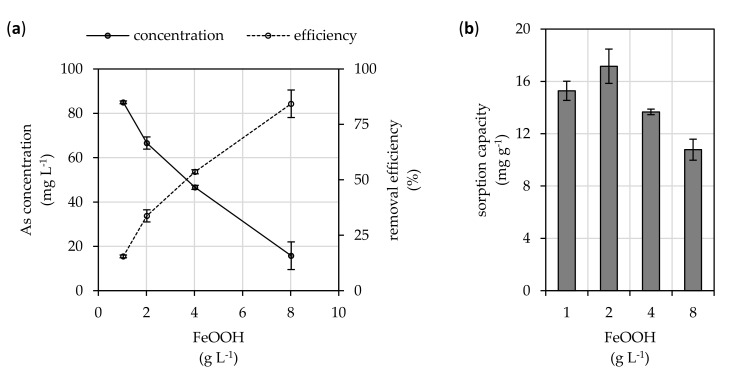
(**a**) Arsenic removal efficiency and its equilibrium concentration in the solution after its sorption onto FeOOH. (**b**) Concentration of arsenic in the FeOOH adsorbent after sorption at equilibrium (initial As concentration was 100 mg L^−1^; contact time 24 h; temperature of 25 °C; agitation rate 130 rpm). Results represent the mean values of four independent experiments, and the error bars show the standard deviation.

Batch adsorption experiments revealed that FeOOH binds arsenate efficiently (Figure 1). As expected, the removed amount of arsenic from the solution by adsorption onto FeOOH increased significantly with the increasing adsorbent dose. Thus, the lowest value of equilibrium arsenic concentration in solution (15.8 mg L^−1^) was observed at the highest adsorbent dose applied (8 g L^−1^). Here, 84% removal efficiency was achieved with 10.8 mg g^−1^ FeOOH sorption capacity for arsenic. However, while the removal efficiency approximated only 34% for a FeOOH dose of 2 g L^−1^, the highest average 17.2 mg g^−1^ sorption capacity was recorded there.

### 3.2. Bioaccumulation of Arsenic

**Figure 2:**
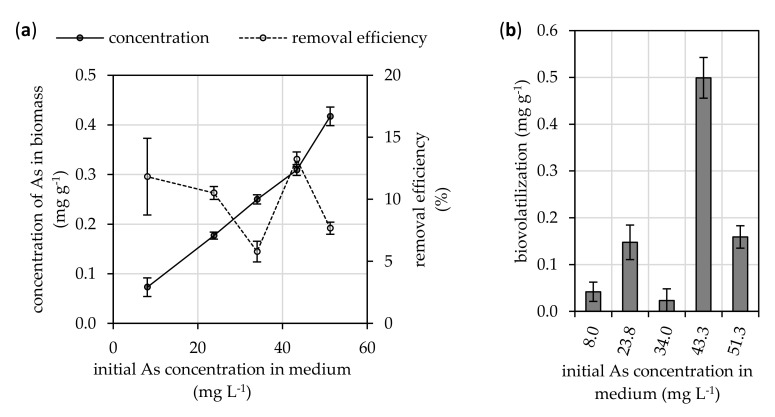
Effects of initial arsenic concentration in a culture medium on its (**a**) bioaccumulation and (**b**) biovolatilization by the fungal *Aspergillus niger* strain during a 19-day static cultivation period at 25 °C. Results represent the mean values of four independent experiments, and the error bars show the standard deviation.

The initial concentration (before the fungus was introduced) of total arsenic (of unknown speciation) in the nutrient medium ranged approximately from 8.0 to 51.3 mg L^−1^. After the growing fungal *A. niger* strain was in contact with the solution for 19 days, the arsenic was partially removed from the culture medium into the biomass (Figure 2a). While there was no general trend in the removal efficiency, the concentration of arsenic bioaccumulated in the fungal biomass increased with initial arsenic content in the culture medium from 0.07 to 0.42 mg g^−1^. Nevertheless, the bioaccumulation efficiency of *A. niger* was relatively low and ranged from 5.8 to 13.2% (Figure 2a).

The contribution of arsenic transformation into volatile derivatives on removal efficiency by fungus was significant (Figure 2b) and accounted for up to a maximum of 0.5 mg g^−1^. This was noted at the initial arsenic concentration of 43.3 mg L^−1^ and it resulted in the highest microbially induced removal of arsenic from the culture medium with an average value of 13.2% of its initial content.

### 3.3. Arsenic Bioextraction

**Figure 3:**
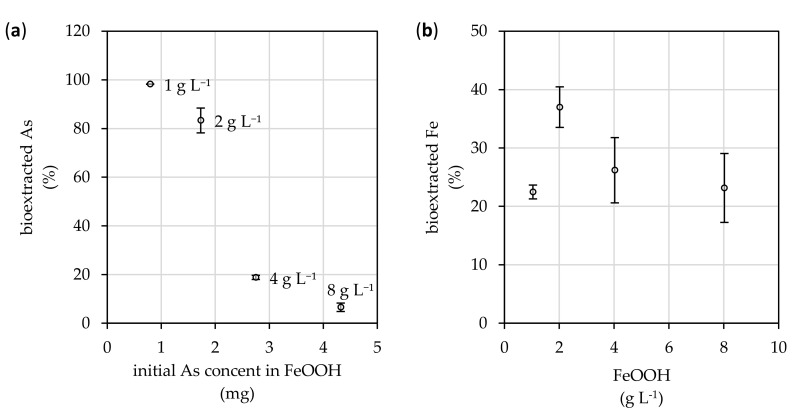
The efficiency of (**a**) arsenic and (**b**) iron bioextraction from the arsenic-treated FeOOH adsorbent after a 19-day exposure period to the fungal *Aspergillus niger* strain during its static cultivation at 25 °C. Results represent the mean values of four independent experiments, and the error bars show the standard deviation.

Before the fungus was inoculated, the initial amount of total arsenic (of unknown speciation) immobilized on the FeOOH surfaces ranged from 0.8 to 4.3 mg. After treatment with growing fungus, the extent of arsenic bioextracted from arsenate-treated FeOOH (indicated by bioextraction efficiency in Figure 3a was primarily attributed to the Fe:As ratio. With increasing iron content in the arsenate-treated FeOOH adsorbent, the arsenic bioextraction efficiency decreased from 98.2 to 6.5%.

**Figure 4:**
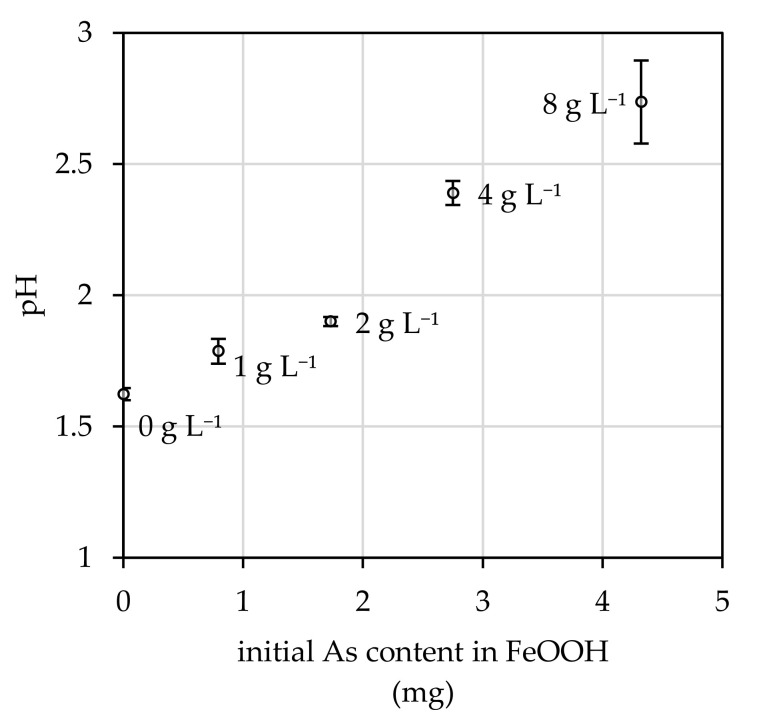
Effect of fungal *Aspergillus niger* 19-day static cultivation period on acidification of culture media supplemented with arsenate-treated FeOOH. Results represent the mean values of four independent experiments, and the error bars show the standard deviation.

The process of bioextraction was triggered by the media acidification due to fungal metabolic activity. Figure 4 highlights that albeit the content of FeOOH suspended in culture medium, the final pH of culture media decreased below the value of 2.7. Still, the presence of arsenate-treated FeOOH suppresses the effect of acidification and, thus, the average value of pH increases from 1.6 to 2.7 with increasing suspended FeOOH content in the medium. Acidification also initiated bioextraction of iron (Figure 3b), which resulted in a significant release of iron from the crystal structure of FeOOH. It was almost 37% at 2.0 g L^−1^, while in the case of other FeOOH doses the bioextraction efficiency approximated 25%.

## 4. Discussion

Iron based phases have been shown to be generally effective adsorbents, which are suitable for removing arsenic from contaminated waters [15]. Special recognition in water treatment is given to amorphous or crystalline ferric oxides or oxyhydroxides due to their high adsorption capacity towards anionic inorganic contaminants and relatively high environmental stability of the formed sorption complexes [26]. The preferential and efficient sorption of arsenate (a dominant anionic specie under oxidative condition of soils, sediments, and surface waters) onto ferric phases is also supported by our experimental data presented in Figure 1. There, at the highest FeOOH doses, the residual concentrations of arsenic in the solution are low (down to 15.8 mg L^−1^), yet, still toxic and significantly over the maximum allowable level of 10 µg L^−1^ [14].

The main reason for FeOOH precipitates’ high affinity towards arsenate and their ability to efficiently remove it by adsorption is the formation of stable complexes through exchange for hydroxyl ligands [27]. However, the exact binding mechanism cannot be proposed, since the FeOOH phase most likely consisted of three different nanosized ferric oxyhydroxides—akaganeite, goethite, and lepidocrocite [12]. Nevertheless, arsenic (V) oxyanions usually form inner sphere mono- and bidentate complexes with goethite via ligand exchange with hydroxyl groups on goethite surfaces [28]. Lepidocrocite also showed the formation of bidentate arsenate complexes [29], and akaganeite preferentially bound arsenic by inner sphere mechanism forming bidentate binuclear complexes between arsenate tetrahedra and the adjacent edge sharing FeO_6_ octahedra [30]. Besides chemisorption, the interaction of arsenic with oxyhydroxides and oxides involves physical adsorption and co-precipitation mechanisms [31]. However, the removal efficiency is also affected by the sorption capacity and limited availability of sorption sites at equilibrium. This manifested in the 15.5% removal efficiency at the lowest 1 g L^−1^ FeOOH dose, while the 15.3 mg g^−1^ sorption capacity there is comparable to capacities at higher adsorbent doses. However, Jacobson and Fan [32] contradicts our finding suggesting that with an increasing amount of goethite (α-FeOOH) in the system, its sorption capacity for arsenic also increases indicating the significance of co-precipitation of arsenic in their experiment. This process is not dependent on the limited sorption sites.

The maximum apparent 17.2 mg g^−1^ sorption capacity is comparable to the results of other authors who used solid phases of FeOOH as adsorbents for arsenate removal. Ladeira and Ciminelli [33] found a maximum sorption capacity of natural goethite for arsenic as high as 12.4 mg g^−1^. Amorphous phases, due to their high active surface area, should have even higher sorption capacities. Dixit and Hering [34] reported that the maximum sorption capacity of amorphous hydrated ferric oxides was up to 157 mg g^−1^. In the form of nanoparticles, the sorption capacity of ferric phases can reach up to 355 mg g^−1^ [35].

However, as previously indicated, the observed arsenic removal after sorption is still insufficient for meeting the requirements of drinking water [36]. Therefore, we attempted to further decontaminate the solution using bioaccumulation by the microscopic filamentous fungus *A. niger*. This strain is ubiquitous in the environment and a common subject of research on water decontamination through bioaccumulation, since it has relatively high resistance to toxic elements [37,38,39,40].

The experimentally determined removal efficiency of up to 13.2% by fungal bioaccumulation is comparable to the experimental data published by Urík et al. [41]. The reported bioaccumulation rate, when the initial concentration of arsenic in the solution was 20 mg L^−1^, was approximately 15.7% by the same fungus. However, other species, such as *Penicillium glabrum*, accumulated up to 93% of arsenate. Therefore, the bioaccumulation efficiency appears to be strain-specific as also highlighted by other authors [42].

Despite the relatively low ability of *A. niger* to bioaccumulate arsenic, the fungus is still interesting in mycoremediation techniques due to its abundance in soils and relatively high ability to adapt to the environment, as well as having high tolerance to various stressors, including the presence of potentially toxic elements [43]. Our strain is also relatively resistant to high concentrations of arsenate in the environment. Dry biomass weight of the fungus decreased only by 15% (data not shown) at the initial arsenic concentration of 51.3 mg L^−1^ in comparison to arsenic-free control. Still, the arsenic exposure was reflected in enhanced arsenic uptake.

The arsenic concentration in the biomass increased linearly with its increasing initial concentration in the culture media (Figure 2a). This indicates that the bioaccumulation capacity of *A. niger* could be much higher than the determined 0.4 mg g^−1^ referred to dry biomass weight and it is highly dependent on the concentration of bioavailable arsenic in the surrounding media. In comparison to active bioaccumulation, Littera et al. [44] suggested that the passive uptake of arsenic by *A. niger* via biosorption is much lower. It accounted for only 0.12 mg g^−1^, although, this was determined for equilibrium arsenic concentrations below 5 mg L^−1^.

The ability of *A. niger* to adapt to arsenic exposure is also manifested in unique metabolic pathway, which leads to arsenic biovolatilization. It was suggested that it may be a fungal detoxification mechanism. In the case of *A. niger*, we assume that the biovolatilization of arsenic takes place intracellularly, which means that bioaccumulation is necessary prior to biovolatilization mechanisms. Therefore, biovolatilization occurs as a mechanism influencing arsenic homeostasis in the biomass. This active mechanism is very effective, and in some cases can intracellularly convert up to 49% of arsenic from a contaminated substrate to a volatile derivatives [45]. The amount of biovolatilized arsenic reached 43.3 mg g^−1^ (Figure 2b), which corresponds to a maximum 62% transformation efficiency of bioaccumulated arsenic by the fungus. This finding is comparable to the ability of various other species of microscopic filamentous fungi to biovolatilize arsenic, such as *Neosartorya fischeri* [46].

In addition to biovolatilization, filamentous fungi possess a unique mechanism that increases the mobility of chemical constituents of solid phases. This relates to the fungal ability to produce active chelating and acidic metabolites that trigger the chemical biodeterioration of solid substrates in the environment via bioextraction [23,39]. It is a part of the natural weathering process of minerals and amorphous phases under natural conditions [47]. Due to its effective use in (bio)technological research [48,49], we have also studied the possible application of bioextraction to recycle arsenic-treated FeOOH using *A. niger* strain.

The amount of bioextracted arsenic is significantly affected by the Fe:As ratio in adsorbent. With increasing iron contents, the efficiency of arsenic bioextraction significantly decreases (Figure 3a). Most of the arsenic was bioextracted into the solution by the fungal metabolites where only 1 g L^−1^ of FeOOH was added, while 6.5% bioextraction efficiency was noted at 8 g L^−1^.

The arsenic bioextraction relates to FeOOH adsorbent’s surfaces interaction with acidic, chelating or redox metabolites of the fungus [50]. It appears that the significant decrease in pH from initial 5.6, caused by fungal metabolites released during cultivation, could be attributed to the extraordinary bioextraction efficiency of *A. niger* strains [38]. As it is depicted in Figure 4, the pH values indicate that with increasing amount of arsenate-treated FeOOH adsorbent in the system, the production of acidic metabolites by the fungus reduces. Still, it is enough to disrupt the FeOOH crystal structure, thereby affecting the release of iron (and arsenic) ions into the culture medium (Figure 3b). This was expected, since it was reported by McDonald et al. [51] that the precipitated ferric phases begin to dissolve naturally when exposed to pH below 3. Nevertheless, since we did not determine the content of either the low molecular weight organic acids or redox active substances produced by the fungus, which may have been involved in the process of FeOOH biodeterioration, the overall importance of pH in the bioextraction of arsenic bound in FeOOH cannot be objectively assessed. Still, in order to recycle the arsenic-treated FeOOH adsorbent, its crystal structure deterioration should be kept at a minimum. Therefore, the noted 37% leaching of iron from FeOOH is the only drawback of this relatively unique and pro-environmental method for adsorbent recycling.

## 5. Conclusions

To conclude our experiment, we assume that the XRD amorphous FeOOH is an effective adsorbent for arsenate removal from contaminated waters. Up to 84% of arsenate was removed from the solution. However, bioaccumulation of the remaining arsenic by *A. niger* did not prove to be an effective post-treatment method for decreasing the arsenic concentration to acceptable levels. Only between 5.8 and 13.2% of the total arsenic in medium was eliminated by the fungus. Subsequently, up to 62% (0.5 mg g^−1^) of bioaccumulated arsenic was transformed into volatile derivatives. In addition to biovolatilization, as an efficient detoxification tool of fungus, *A. niger* was capable of extracting arsenic and iron from FeOOH adsorbent due to the significant acidification of the media. While the bioextraction of iron was relatively high (up to 37%), the arsenic removal from the FeOOH adsorbent was successful with up to 98% bioextraction efficiency. This shows that the natural biodeterioration of the FeOOH surfaces by fungus is capable of increasing reusability of this efficient arsenate adsorbent, and it indicates the applicability of the fungal *A. niger* strain in techniques that can increase the cost-effectiveness of pro-environmental remediation processes.
